# *Trypanosoma cruzi* burden, genotypes, and clinical evaluation of Chilean patients with chronic Chagas cardiopathy

**DOI:** 10.1007/s00436-015-4503-2

**Published:** 2015-05-03

**Authors:** Werner Apt, Arturo Arribada, Inés Zulantay, Miguel Saavedra, Eduardo Araya, Aldo Solari, Sylvia Ortiz, Katherine Arriagada, Jorge Rodríguez

**Affiliations:** Laboratorio de Parasitología Básico-Clínico, Programa de Biología Celular y Molecular, Instituto de Ciencias Biomédicas, Facultad de Medicina, Universidad de Chile, Independencia 1027, PO 9183, Santiago 1, Chile; Clínica INDISA, Santiago, Chile; Laboratorio de Biología Molecular de Parásitos, Programa de Biología Celular y Molecular, Instituto de Ciencias Biomédicas, Facultad de Medicina, Universidad de Chile, Santiago, Chile; Escuela de Salud Pública, Facultad de Medicina, Universidad de Chile, Santiago, Chile

**Keywords:** Chagasic cardiopaths, *Trypanosoma cruzi* DTUs, Parasite burden, Genotypes

## Abstract

There are currently no biomarkers to assess which patients with chronic indeterminate Chagas disease will develop heart disease and which will spend their entire life in this state. We hypothetize that the parasite burden and *Trypanosoma cruzi* genotypes are related to the presence of heart disease in patients with Chagas disease. This study is aimed to investigate the parasite burden and *T. cruzi* genotypes in chagasic cardiopaths *versus* chagasic individuals without cardiac involvement according to the New York Heart Association. Patients with chronic Chagas disease, 50 with and 50 without cardiopathy (controls), groups A and B, respectively, were submitted to anamnesis, physical examination, and electrocardiogram. Echo-Doppler was performed for group A; all important known causes of cardiopathy were discarded. Xenodiagnosis, conventional PCR, and quantitative PCR were performed on patients of both groups. *T. cruzi* genotyping was done for 25 patients of group A and 20 of group B. The 50 cardiopaths had 80 electrocardiographic alterations, most of them in grade II of the New York Heart Association classification; 49 were classified in grade I by Echo-Doppler, and only one patient was in grade III. The difference in average parasitemia in patients of groups A and B was not significant. The most frequent *T. cruzi* DTU found was TcV*.* The parasite burden and genotype of the groups with and without cardiopathy were similar.

**Graphical abstract:**
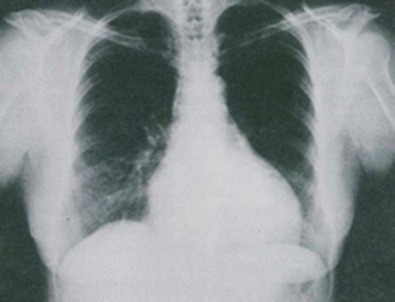
Imagen 1 Chronic chagas cardiopathy chest X-ray heart enlargement

Imagen 1 Chronic chagas cardiopathy chest X-ray heart enlargement

Figure 2 Chronic Chagas cardiopathy microaneurism of left ventricle. Cineangiography

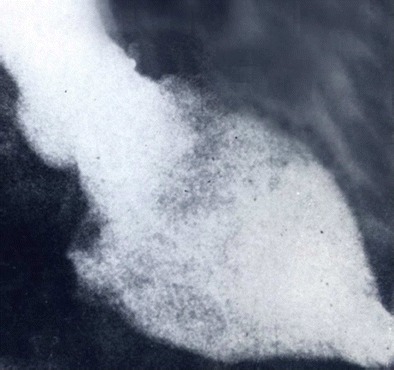

## Introduction

Chagas disease (ChD) is a pathology that affects about 6–7 million people in the American continent (WHO 2015) where it is autochthonous and has been introduced into Europe, Asia, and Oceania through human migration (Basile et al. 2011; Molina-Berríos et al. 2013). There are 80–100 million people at risk of acquiring the disease (Salvatella 2012).

ChD is currently one of the most important neglected diseases. It represents the fourth disease in importance in years lost due to incapacity (Tarleton and Curran 2012; Apt et al. 2013). Two periods characterize the natural evolution of the disease, acute and chronic; the latter with persistent infection in the heart and adipose tissue (Wen et al. 2014) may be latent, indeterminate, or determinate (Coura and Viñas 2010; Rassi et al. 2010). Less than 5 % of the individuals in the acute period develop symptomatology, especially in children. Fifty to seventy percent of chronic chagasic cases have a latent or indeterminate stage which lacks clinical symptoms and with normal routine clinical laboratory tests; 10–30 % develop cardiac pathology and 8–10 % digestive commitment. Without doubt, chronic Chagas cardiopathy is the worst form of the disease, due to its morbidity and mortality (Rassi et al. 2010). Young people with ChD and normal ECG have periods of survival nine times greater than chagasic persons of the same age with an abnormal electrocardiogram (Rassi et al. 2009). Patients with cardiomyopathy due to *Trypanosoma cruzi* and cardiac insufficiency have worse prognosis than those heart myopathies with decompensated heart failure due to other aetiologies (69.2 v/s 47.9 % mortality at 1 year, respectively) (Silva et al. 2008). The outcome of Chagas cardiomyopathy is worse than idiopathic cardiomyopathy (Barbosa et al. 2011). Chagas cardiomyopathy presents a higher frequency of stroke compared to a non-Chagas cohort (Da Matta et al. 2012). The annual cost of Chagas cardiomyopathy, including both health costs and disability-adjusted annual losses, exceeds that which is originated by cervical uterine cancer and rotavirus, according to simulated computer models (Lee et al. 2013). About 50–60 % of the people with the indeterminate form remain in this asymptomatic state for life. For example, Berenice, the first patient of Carlos Chagas (discoverer of the disease) whose ChD diagnosis was performed at age of two, died at 82 years of age without presenting any symptomatology due to ChD in her life. In Brazil and Chile, 1–2 % of the patients with indeterminate chronic ChD develop cardiopathy each year.

It is very important to know which infected subjects will develop heart disease and who will not, to apply etiologic therapy to the former and not to all the patients with indeterminate chronic ChD. To date, no biomarkers are available to answer this question (Venegas et al. 2010; Requena-Méndez et al. 2013; Urbina 2015). To determine whether the parasite burden and *T. cruzi* genotypes are related to the presence of heart disease in patients with ChD, we conducted this study aimed to investigate the parasite burden and *T. cruzi* discrete typing units (DTUs) TcI-TcVI in chagasic cardiopaths *versus* chagasic individuals without cardiac involvement according to the New York Heart Association (NYHA) (1994; Cura et al. 2012).

## Material and methods

### Population

Adult patients with chronic ChD of the IV region (Coquimbo), Chile from the localities of Illapel and Salamanca (Choapa province) and Combarbalá (Limarí province), hyperendemic areas located between 29° 02´ and 32° 16´ South latitude, were examined twice a year in outpatient clinics (rural patients) and hospitals (urban patients) by our investigation group. They were submitted to anamnesis, physical examination, and ECG of twelve leads. The patients were divided into two groups according to whether or not they presented ECG alterations. Fifty individuals with chronic chagasic cardiopathy were randomly selected (group A) and 50 without cardiopathy (group B). Echo-Doppler was performed on the patients of group A to eliminate other important known causes of cardiopathy, hypertension, valve disease, atherosclerosis, myocardiopathy, and congenital malformations. Xenodiagnosis (XD), conventional PCR (cPCR), and quantitative PCR (qPCR) for *T. cruzi* were performed on patients of both groups. Genotyping of *T. cruzi* was successful in 25 patients of group A and 20 of group B.

### Ethics statement

The participation of the patients was under Informed Consent approved by the Ethical Committee of the Faculty of Medicine of the University of Chile (Protocol No. 048-11). Informed consent from patients was given in written form.

### Conventional serology

In the IIF test, epimastigote forms of *T. cruzi*, Tulahuén strain, were used as antigens. They were cultured in Diamond medium supplemented with 5 % fetal bovine serum maintained at 28 °C. In the exponential growth phase, parasites were collected by centrifugation at 1300*g* for 10 min at 4 °C (Maya et al. 2007). Titers equal or superior to 1/20 dilutions were considered positive. In each determination, positive and negative controls from chagasic and non-chagasic individuals were included. An ELISA test was applied using Chagatek ELISA (BioMerieux, France). The optical density corresponding to the cutoff value was determined by the average of the negative control plus 0.100. The ELISA OD values for the negative controls in the different assays fluctuated between 0.001 and 0.09. The plate was read in a spectrophotometer PHOMO of Autobio, by indications of Micro-Elisa System.

### Conventional xenodiagnosis

The colony of *Triatoma infestans* used in xenodiagnosis (XD) has been maintained in our laboratory for 50 years fed on chickens, which are normally refractory to *T. cruzi* (Schenone 1999). XD was applied using two cylindrical wooden boxes each containing seven uninfected third or fourth instar nymphs of *T. infestans* starved for a period of 3–4 weeks. The insects of the cages were fed by patients and then maintained at 27 °C and 70 % relative humidity without further feeding. After 30, 60, and 90 days of incubation, the rectal contents of triatomines fed on each patient were obtained by slight abdominal compression in a biologically secure hood, for examination under an optical microscope at ×40, 100 fields were observed to detect mobile trypomastigotes or epimastigotes of *T. cruzi.* The criteria to determine a negative XD was the absence of mobile forms of *T. cruzi* in the three periods of microscopic observation (30, 60, and 90 days) (Saavedra et al. 2013).

### Electrocardiographic tracing

The patients were evaluated by a twelve-lead electrocardiogram. Each electrocardiographic trace included the following parameters: P axis, P duration, P-R space, R-R space, R space, QT value, QTc calculation, QRS axis, T axis, ventricular gradients, RV1 intrensicoide deflexion, SV1, RVS, Sokolow index, and an electrocardiographic diagnosis. The final interpretation of this test data was performed by a specialist cardiologist following the double blind protocol recommended by the World Health Organization; the investigator analyzing the ECG traces was unaware of the status of the patients (Maguire et al. 1982).

### Echo-Doppler

The Echo-Doppler was performed in Baquedano Square Medical Image Centre of Santiago, Metropolitan Region, Chile, with a latest generation Philips apparatus. The following parameters were measured with bi-dimensional M mode: systolic diameter of left ventricle, diastolic diameter of left ventricle, septum of left ventricle, posterior wall of left ventricle, left auricular size, hypertrophic sign of left ventricle, mass of left ventricle, and ejection fraction of left ventricle. The Doppler measurements allowed the determination of the status of the different valves and the presence of reflux. The final interpretation of this test was performed by a cardiologist specialist in echography.

### DNA extraction

Five milliliters of venous blood of each patient was mixed with the same volume of a 6 M guanidine hydrochloride 0.2 M EDTA pH 8.0 solution, incubated at 98 °C for 15 min to nick DNA of *T. cruzi* minicircles and stored at 4 °C. DNA extraction was performed in 200 μL of the samples mixture, using the QIAamp® DNA Blood Mini Kit (Qiagen, Valencia, CA) according to the manufacturer`s instructions. The purified DNA was maintained at −20 °C until use.

### Conventional PCR

Conventional PCR (cPCR) was performed in triplicate using oligonucleotides 121 and 122, which anneal to the four conserved regions present in *T. cruzi* minicircles (Degrave et al. 1988), to obtain 330 bp amplicons. Each sample was tested in a final volume of 20 μL including 5 μL of extracted DNA. The final concentrations of the reagents were as follows: 2.5 mM MgCl_2_, 0.2 mM of each dNTP, 0.5 μM of each primer, and 1 unit GoTaq DNA polymerase (Promega Corp., USA). The amplification program was performed in a TC-412 thermal cycler (Techne, UK) which included an initial denaturation at 98 °C for 1 min and 64 °C for 2 min; 33 cycles of 94 °C for 1 min, 64 °C for 1 min and 72 °C for 1 min, and a final extension at 72 °C for 10 min. Each experiment included two negative PCR controls: water instead of DNA and DNA of non-chagasic patients. As positive control purified DNA of *T. cruzi* Tulahuén strain was used. Amplification products were analyzed by electrophoresis in a 2 % agarose gel and visualized after staining with RedGel (Biotium Inc.). Five microliter Bench Top 100 bp DNA ladder (Promega Corp.,USA) was incorporated. A positive result for cPCR was the presence of a 330 bp band specific for *T. cruzi* minicircles.

### Quantitative PCR for *Trypanosoma cruzi* (qPCR)

The TaqMan® detection system was applied in a Stratagene MX3000P^TM^ thermocycler (Agilent Technologies) under conditions suggested by the manufacturer and using primers of DNA satellite Cruzi 1, Cruzi 2, and Cruzi 3 (Schijman et al. 2011). The reaction mixture consisted of 2 μl of the sample to be investigated, 10 μl Brilliant Multiplex QPCR Master mix (Stratagene), 0.5 μl of a 1:500 dilution of a reference dye (ROX), 0.5 μl each of Cruzi 1, and Cruzi 2, 0.2 μl Cruzi 3, 0.2 μl BSA (100x) and 6.1 μl Molecular Biology Grade Water (Mo Bio Laboratories Inc.) in a final volume of 20 μl. To obtain a standard curve to perform the quantification, we used a stock of epimastigote forms of *T. cruzi*, Tulahuen strain. The total DNA quantification was carried out using AccueBlue^™^ High Sensitivity dsDNA Quantitation kit (Biotum Inc.) and the qPCR instrument Mx3000P ^™^ (Stratagene, Agilent Technologies Inc.) as detector devices according to Bravo et al. (2012). A *T. cruzi* DNA concentration equivalent to 1×10^6^ epimastigotes/ml was adjusted, considering that 1 parasite cell contains 200 fg of DNA(Duffy et al. 2009). The DNA was diluted 1:10. The standard curve of *T. cruzi* was maintained at −20 °C until use.

The controls used for qPCR-*T. cruzi* were as follows: negative control, DNA of a non-chagasic patient confirmed by serology (IIF and ELISA), evaluated previously with qPCR equipment and positive controls, DNA of an individual with ChD with confirmed parasitemia by PCR and evaluated previously in qPCR equipment. Control mixture: 20 μl of mixture reaction for *T. cruzi* (without the sample under study). Water control: 2 μl of water free of nucleases (Mo Bio Laboratories Inc.) (replacing the study sample). The control mixture and water control are useful to evaluate contamination in the preparation of the mixture reaction or unspecific qPCR reactions. Each point of the standard curve was performed in triplicate. The samples and controls were included in duplicate. As an internal control of extraction and inhibition of qPCR, we used chromosome 12 (X12) (Bravo et al. 2012). X12 primers were designed by N. Jullien using the AmplifX v.1.5.4 software, and compared with Nucleotide BLAST (National Library of Medicine) to discount any other unspecific amplification (N. Nazal, personal communication). The N1X12 forward (5′-GCTGGCTAGACTGTCAT-3′) and N2X12 reverse (5′-CTTTGCCGTTGAAGCTTG-3′) and N3X12 probe (N3X12 5′-/56-FAM/TGGGACTTC/ZEN/AGAGTAGGCAGATCG/3IABkFQ/-3′) primers were used. The standard curve for X12 was prepared with a pool of human genomic DNA of five non-chagasic individuals diluted 1/5 in elution buffer. The reaction mixture was composed of 10 μl Brilliant Multiplex QPCR Master Mix, 0.5 μl of a 1:500 solution of Reference Dye (ROX), 2 μl each N1/N2, 0.8 μl N3, 0.2 μl BSA (100x), 2.5 μl Molecular Biology Grade water (Mo Bio Laboratories, Inc.), and 2 μl of DNA isolate in a final reaction volume of 20 μl. The thermals profiles of qPCR-*T. cruzi* and qPCR-X12 included 10 min of pre-incubation at 95 °C and 40 amplification cycles (95 °C for 15 s, 60 °C for 1 min). The measurement of emitted fluorescence was performed at 60 °C at the end of each cycle. The MxPro v4.1 (Agilent Technologies) software delivered automatically the parasites/ml data.

### Hybridization assays

*T. cruzi* genotyping was performed twice by DNA blot analysis of DNA minicircle amplicons in 75 patients. In 21 of them, the concentration of PCR products was below the minimum required for hybridization tests. The primers for probe generation were CV1 (5′-GATTGGGGTTGGAGTACTAT-3′) and CV2 (5′-TTGAACGGCCCTCCGAAAAC-3′), which produced a 270-bp fragment. These fragments were further digested with restriction endonucleases Sau96I and ScaI (Fermentas, Ontario, Canada) to obtain a 250-bp band and remove all sequences from the minicircle constant region. Finally, the probes sp104c11 (TcI), CBBc13 (TcII), NRc13 (TcV), and v195cl1 (TcVI) were labeled using the random primer method with [α-^32^P] dATP. The membranes were pre-hybridized in a solution of 6×SSC and 1 mM EDTA pH 7.4 with salmon sperm DNA at 55 °C. The membranes were hybridized overnight with the labeled probe at 55 °C in the same solution and then washed in high-stringency conditions (0.1×SSC and 0.1×SDS at 68 °C). The membranes were exposed for 1–4 h in a Molecular Imager (FX Bio Rad Laboratories, Hercules, CA). This method has been validated by hybridization with the probes constructed by PCR amplification of *T. cruzi* DNA from different kDNA DTU typing (Veas et al. 1991; Brenière et al. 1995, 1998; Solari et al. 2001; Rodríguez et al. 2009; Arenas et al. 2012). The hybridization profiles were analyzed to compare the intensity of the ethidium bromide-stained bands in each membrane with the presence of the radioactive bands obtained with each probe. The specificity of the probes was tested in hybridization controls against DNA of the probes themselves. The profiles of radioactive hybridization signals with multiple probes are indicative of mixed infections with several genotypes of *T. cruzi.*

### Statistical analysis

The data were analyzed using the SPSS program version 19.0. The description of the data was performed by tables, arithmetic mean, mode, standard deviation, and amplitude of the variable. Chi square was applied for group comparison of qualitative variables. In the case of quantitative variables, Levene tests were applied to evaluate the homoscedasticity and Student’s *t* test to evaluate the average burden. A significance level of 0.05 was used.

## Results

### Age, gender, and geographical origin of groups A and B

Ages of the individuals of group A ranged from 30 to 81 years, average 58.3. Sixty percent of the patients were women. Most patients were from Combarbalá (58 %), and 70 % lived in rural zones. In group B, the average age was 49.6 years, ranging from 20 to 76 years old. Eighty percent of the patients were women. Most patients were from Illapel (42 %), and 66 % lived in rural zones. The patients of groups A and B were examined in the outpatient clinics and hospitals where they live between 2011 and 2013. The average age of group A is significantly greater that of group B (*t* = 3.39; *p* = 0.001) (Tables 1 and 2).

**Table 1:** Clinical, epidemiological, serological, parasitological, comorbidity, electrocardiographical, and Echo-Doppler characteristics of 50 chronic chagasic cardiopaths. Group A

No.	Age	Sex	Locality	Origin		Control date	Serology		cPCR	qPCR		Xenodiagnosis	DTU *T. cruzi*				ECG	NYHA group	Comorbidity
				Urban	Rural		IIF-IgG	ELISA D.O.		p/ml	Ct		Tcl	Tcll	TcV	TcVl			
1	73	F	Salamanca		x	01-06-2011	+1/640	1.628	+	0.37	35.77	-					LVH, LAHB	II by ECG	Hypertension, depression, diabetes mellitus, hypercholesterolemia, hypothyroidism
2	55	M	Combarbalá		x	06-07-2011	+1/1280	2.507	+	0.54	35.18	+			+		SB	I by ECG	Cholecystectomy
3	56	F	Combarbalá		x	06-07-2011	+1/640	1.568	+	0.4	35.67	+			+		IRBBB	I by ECG	
4	49	M	Illapel		x	06-07-2011	+1/640	1.905	+	0.17	36.46	−			+		QTc↑, VE, LAHB, IRBBB	II by ECG	Hypertriglyceridemia
5	60	M	Salamanca	x		10-01-2011	+1/640	1.974	−	0	No Ct	−					LAHB	II by ECG	Hypertriglyceridemia
6	59	M	Salamanca		x	13-01-2011	+1/640	1.413	+	0	No Ct	−				+	SB	I by ECG	
7	54	F	Combarbalá	x		01-08-2011	+1/40	0.544	+	0.4	35.67	−					QTc↑, LAHB	II by ECG	Hypertension, hypercholesterolemia, prediabetes
8	47	F	Combarbalá		x	01-08-2011	+1/640	1.313	+	2.38	32.99	−	+	+	+		QTc↑	II by ECG	Hypertension, cholecystectomy, depression, gastritis
9	60	F	Combarbalá		x	01-08-2011	+1/640	1.356	+	0.34	35.88	−					QTc↑, LAHB	II by ECG	Gastritis, depression
10	70	M	Combarbalá	x		01-08-2011	+1/1280	2.059	+	0.39	35.70	−		+	+	+	CRBBB	II by ECG	Hypertension, arthrosis
11	43	F	Combarbalá		x	27-08-2011	+1/1280	2.176	+	0.38	35.74	−					LPHB, CRBBB	II by ECG	
12	48	M	Combarbalá		x	19-01-2011	+1/1280	2.022	+	0.53	35.22	−					SB, LVH	II by ECG	Esophagus achalasia
13	69	F	Combarbalá		x	01-08-2011	+1/640	1.403	+	0.49	35.36	−		+	+	+	LAHB	II by ECG	Hypertension
14	71	F	Illapel	x		01-09-2011	+1/640	1.225	+	0.8	35.76	−					SB, LAHB	II by ECG	Hypertension, hypercholesterolemia
15	77	F	Illapel	x		01-09-2011	+1/640	2.099	+	0.44	35.53	-			+		AE, LAHB, ischemia	II by ECG	Hypertension, prediabetes, cholecystectomy
16	55	M	Combarbalá	x		01-07-2012	+1/1280	1.987	+	0.05	38.54	+			+		I°st AV block	II by ECG	
17	50	F	Combarbalá		x	01-07-2012	+	1/1280	1.719	+	0.57	35.08	−				QTc↑, SB, I°st AV block	II by ECG	
18	79	F	Combarbalá	x	01-07-2012	+	1/640	1.837	+	0.1	36.71	−			+		QTc↑ SB, I°st AV Block	II by ECG	Cholecystectomy
19	52	F	Combarbalá		x	01-07-2012	+1/1280	1.831	−	0	No Ct	−					SB	I by ECG	
20	77	F	Combarbalá		x	01-07-2012	+1/640	1.589	+	0	No Ct	−			+		QTc↑, SB, I°st AV block, LVH	I by ECG	Hypertension, cholecystectomy
21^a^	68	M	Combarbalá		x	01-07-2012	+1/1280	1.734	+	0.49	35.36	+			+		SB	I by ECG o III by EF	Hypertension
22	60	F	Illapel	x		23-06-2011	+1/1280	1.974	+	0.38	35.74	−					SB, Anterior ischemia	II by ECG	Scoliosis, depression
23	53	F	Illapel	x		01-07-2012	+1/1280	2.037	+	0	No Ct	−			+		QTc↑, repolarization alteration	II by ECG	Hypertension
24	30	F	Illapel	x		01-09-2011	+1/640	1.179	+	0.58	35.05	−					QTc↑, VE	II by ECG	Hypothyroidism
25	53	M	Salamanca		x	01-04-2013	+1/160	0.485	+	0.26	36.15	+					SB	I by ECG	
26	57	M	Combarbalá	x		01-07-2012	+1/320	1.641	+	0.3	36.01	−	+		+	+	SB	I by ECG	
27	75	F	Illapel		x	01-07-2012	+1/1280	2.068	+	0.01	39.92	−			+	+	QTc↑, VE	II by ECG	Hypertension, cholecystectomy
28	53	F	Combarbalá		x	01-07-2012	+1/640	1.986	+	0	No Ct	−			+		SB	I by ECG	Bronchial asthma
29	72	F	Combarbalá		x	01-07-2012	+1/80	1.331	+	0.07	37.84	−			+		Repolarization alteration	II by ECG	Cholecystectomy
30	64	M	Combarbalá	x		01-07-2012	+1/640	1.603	−	0.01	39.92	−					LVH	II by ECG	Hypertension
31	41	F	Illapel		x	01-07-2012	+1/80	1.415	+	0.01	39.92	−	+		+		QTc↑, SB	I by ECG	Hypercholesterolemia
32	47	M	Illapel		x	01-07-2012	+1/640	1.776	+	0.01	39.92	−		+			QTc↑, LAHB, CRBBB	II by ECG	Hypercholesterolemia
33	31	F	Illapel		x	20-05-2011	+1/1280	1.552	+	0.36	35.81	−			+		QTc↑	II by ECG	
34	76	M	Illapel	x		01-07-2012	+1/1280	2.379	+	2.1	33.09	+			+		Repolarization alteration	II by ECG	Hypertension, gastritis, severe hearing loss
35	81	F	Illapel		x	01-07-2012	+1/320	1.121	−	0	No Ct	−					SB	I by ECG	Hypertension, prediabetes, hypercholesterolemia
36	60	F	Illapel		x	01-04-2013	+1/1208	2.327	+	0.51	35.29	−			+		SB	I by ECG	
37	70	F	Illapel		x	01-04-2013	+1/640	1.371	+	0.19	36.39	−		+		+	Lateral ischemia	II by ECG	Hypertension, bronchial asthma
38	67	F	Combarbalá		x	01-10-2012	+1/320	1.393	+	0.32	35.94	+				+	AE	II by ECG	Hypertension, hypercholesterolemia, hypothyroidism, depression
39	38	M	Illapel		x	01-01-2011	+1/1280	2.506	+	0.18	36.43	−			+		QTc↑, SB	I by ECG	
40	56	M	Illapel	x		01-08-2013	+1/160	0.784	+	0.06	38.19	−					SB	I by ECG	Hypercholesterolemia
41	55	F	Combarbalá		x	06-06-2013	+1/640	1.541	−	0.009	39.99	−					SB	I by ECG	Hypertension, hypothyroidism, hypercholesterolemia
42	59	M	Combarbalá		x	01-08-2013	+1/640	1.871	+	0.13	36.60	+					SB	I by ECG	
43	71	M	Combarbalá		x	01-08-2013	+1/320	1.315	−	0.04	38.88	+					SB	I by ECG	Hypertension
44	51	F	Combarbalá		x	01-07-2012	+1/160	0.362	+	0.15	36.53	−					QTc↑	II by ECG	
45	72	F	Combarbalá		x	01-08-2013	+1/640	1.754	−	0.02	39.57	−					SB	I by ECG	Hypertension, hypercholesterolemia
46	38	M	Combarbalá		x	06-06-2013	+1/320	1.060	−	0.02	39.57	+					SB	I by ECG	Prediabetes
47	49	F	Combarbalá	x		01-06-2013	+1/80	1.319	+	0.05	38.54	−					LAHB	II by ECG	
48	70	F	Combarbalá	x		01-11-2013	+1/320	1.358	−	0.03	39.23	−					QTc↑, CRBBB	II by ECG	
49	48	M	Combarbalá		x	01-11-2013	+1/640	1.651	−	0.03	39.23	−					QTc↑, CRBBB	II by ECG	
50	46	M	Salamanca	x		01-11-2013	+1/1280	1.483	+	0.02	39.57	+					IRBBB	I by ECG	Hypertension, arthrosis, cholecystectomy

*LVH* left ventricle hypertrophy, *LAHB* left anterior hemiblock, *SB* sinus bradycardia, *IRBBB* incomplete right bundle branch block, *QTc↑* prolongued QTc interval, *VE* ventricular extrasystoles, *CRBBB* complete right bundle branch block, *LPHB* left posterior hemiblock, *AE* auricular extrasystoles, *EF* ejection fraction

^a^This case presents low ejection fraction by Echo-Doppler

**Table 2:** Clinical epidemiological, serological, parasitological, and comorbidity of 50 chronic chagasic patients without cardiopathy. Group B

No.	Age	Sex	Locality	Origin		Control date	Serology		cPCR	qPCR		Xenodiagnosis	DTU *T. cruzi*				Comorbidity
				Urban	Rural		IIF-IgG	ELISA D.O.		p/ml	Ct		Tcl	Tcll	TcV	TcV1	
51	43	M	Combarbalá	x		01-07-2012	+1/640	2.106	+	0.03	39.23	–					
52	32	F	Illapel		x	01-09-2011	+1/1280	1.952	+	0.37	35.77	–			+		
53	76	F	Combarbalá		x	01-07-2012	+1/320	1.375	+	0.4	35.67	+					Diabetes, hypercholesterolemia, cataract
54	38	F	Illapel	x		01-09-2011	+1/80	1.692	+	1.13	33.43	–			+		
55	66	F	Salamanca		x	01-07-2012	+1/320	0.685	+	0.02	39.57	−					Esophagus achalasia, hypothyroidism, chronic gastritis
56	30	F	Illapel	x		01-07-2012	+1/640	1.785	−	0	No Ct	–					
57	32	M	Illapel		x	01-01-2011	+1/1280	1.784	+	0.03	39.23	+					
58	56	F	Illapel	x		01-07-2012	+1/1280	2.114	+	0.29	36.05	+			+		Cholecystectomy
59	21	M	Salamanca		x	29-08-2011	+1/40	1.302	+	0	No Ct	−	+			+	
60	57	F	Illapel	x		01-07-2012	+1/320	1.085	−	0	No Ct	−					Hypertension, cholecystectomy, hypercholesterolemia,
61	56	F	Salamanca		x	13-01-2011	+1/640	1.804	−	0	No Ct	−					Hypertension, cholecystectomy, arthrosis
62	61	F	Illapel	x		01-01-2011	+1/1280	2.196	−	0.01	39.92	–					
63	34	F	Illapel	x		01-07-2012	+1/320	1.090	+	0.06	38.19	−					Bronchial asthma
64	44	F	Salamanca		x	01-07-2012	+1/1280	2.248	−	0	No Ct	−					Hypertension
65	53	F	Salamanca		x	01-07-2012	+1/640	1.726	+	0	No Ct	−				+	Hypertension
66	49	M	Combarbalá		x	01-01-2011	+1/1280	2.281	+	0.03	39.23	–				+	
67	20	F	Illapel		x	01-07-2012	+1/1280	2.288	+	0.13	36.60	−					
78	56	F	Illapel	x		01-07-2012	+1/1280	2.240	+	0.01	39.92	−		+			Hypertension, gastric ulcer
69	61	M	Salamanca	x		01-07-2012	+1/320	1.370	+	0.01	39.92	−		+	+	+	Colon cancer
70	62	F	Illapel	x		01-09-2011	+1/1280	1.654	+	0.5	35.32	−					Hypertension, arthrosis, hypothyroidism
71	55	F	Salamanca		x	13-01-2011	+1/1280	2.276	+	1.04	33.46	+					
72	65	M	Salamanca		x	01-07-2011	+1/40	1.495	+	1.27	33.38	−					Hypertension
73	60	F	Combarbalá		x	01-07-2012	+1/1280	2.575	+	0	No Ct	−			+		Cholecystectomy, hydatidosis
74	63	F	Combarbalá		x	01-08-2011	+1/1280	2.150	+	1.04	33.46	+			+		
75	47	F	Combarbalá		x	01-07-2012	+1/160	1.470	+	0.02	39.57	−			+		
76	61	F	Combarbalá		x	01-08-2011	+1/640	1.673	+	0.33	35.91	−					Cholecystectomy
77	42	F	Combarbalá		x	01-07-2012	+1/640	1.645	+	0.06	38.19	−			+		
78	58	F	Illapel		x	01-11-2013	+1/1280	1.633	+	0.39	35.07	−					Hypertension, hypothyroidism
79	56	M	Illapel	x		01-11-2013	+1/640	1.817	−	0.02	39.57	+					
80	55	M	Illapel	x		01-11-2013	+1/640	1.796	−	0	No Ct	−					Hypertension, bronchial asthma
81	43	F	Illapel	x		01-11-2013	+1/320	1.717	+	0.34	35.88	−			+		
82	30	F	Illapel	x		01-11-2013	+1/320	1.195	−	0	No Ct	−					
83	49	F	Salamanca		x	01-11-2013	+1/640	1.503	−	0.03	39.23	−					Hypertension, cholecystectomy
84	72	F	Salamanca		x	01-11-2013	+1/1280	1.629	−	0	No Ct	−					Hypertension, diabetes mellitus, cerebellar ataxia
85	58	F	Combarbalá		x	01-11-2013	+1/640	1.772	+	0.04	38.88	−					Hypertension, hypercholesterolemia
86	51	F	Combarbalá	x		01-11-2013	+1/160	0.743	−	0	No Ct	−					
87	42	F	Illapel		x	30-08-2011	+1/160	0.634	−	0	No Ct	−					
88	33	F	Combarbalá		x	01-08-2011	+1/320	1.478.	+	0.1	36.71	+					
89	53	F	Salamanca		x	01-07-2011	+1/1280	1.635	+	0.5	35.32	+					
90	38	F	Salamanca		x	01-07-2011	+1/1280	1.689	−	0	No Ct	–					
91	60	F	Illapel		x	01-07-2012	+1/640	1.441	−	0.02	39.57	–					Hypertension
92	65	F	Combarbalá	x		01-07-2012	+1/1280	1.944	+	0.07	37.84	−					Cholecystectomy
93	41	F	Illapel		x	01-07-2012	+1/640	1.602	+	0	No Ct	−			+		Fibromyomas, nephrolithiasis
94	52	M	Combarbalá		x	06-07-2011	+1/1280	2.337	+	0.59	35.01	+	+	+	+		Esophagus achalasia
95	57	F	Illapel		x	01-14-2013	+1/40	1.094	+	0.04	38.88	−			+		Cholecystectomy, hypercholesterolemia
96	48	M	Combarbalá		x	01-07-2012	+1/320	1.347	+	0.04	38.88	−		+			Megacolon
97	46	F	Combarbalá		x	01-06-2013	+1/160	0.411	+	0.37	35.77	−				+	Cholecystectomy
98	20	F	Salamanca		x	01-10-2012	+1/160	0.549	+	0.67	34.73	+			+	+	
99	50	F	Salamanca	x		01-04-2013	+1/640	1.539	−	0	No Ct	-					
100	62	F	Illapel		x	01-07-2012	+1/320	0.862	+	0.02	39.57	−			+	+	Osteoporosis

### Serological results

The titers of the IIF IgG were between 1/40 and 1/1280, and the OD of the ELISA was 0.17 to 2.575. The OD values from the ELISA and titers from the IIF tests demonstrated broad agreement for patients exhibiting both strong and weak serological responses, in agreement with previous studies (Umezawa et al. 1996; Luquetti et al. 2009) (Tables 1 and 2).

### Parasite burden and genotypes

The averages obtained in the standard curves of qPCR for *T. cruzi* for the parameters were as follows: correlation coefficient (*R*^2^) 1.00, slope (Y) 3.46, and efficiency (Eff) 94.45. The Ct averages for each point of the standard curve were as follows: 10^5^ parasites/ml Ct: 16.59; 10^4^ parasites/ml Ct: 19.97; 10^3^ parasites/ml Ct: 23.47; 10^2^ parasites/ml Ct: 27.11; 10^1^ parasites/ml Ct: 30.65; 1 parasites/ml Ct: 33.47; 10^−1^ parasites/ml Ct: 36.71. In the cases of group A, the parasite burden fluctuated between 32.99 and 39.99, corresponding to 2.38 and 0.009 parasites/ml, respectively. The average Ct in the 50 patients of this group was 36.88. In the cases of Group B, Ct fluctuated between 33.88 and 39.92, corresponding to 1.27 and 0.01 parasites/ml, respectively. The average Ct in the 50 patients of this group was 37.23. In the standard curve, the last point corresponds to 0.1 parasites/ml. Automatic interpolation occurred where the *T. cruzi* parasitemia estimated by qPCR was less than 0.1 parasites/ml using the MxPro v4.1 (Agilent Technologies) software package. Average levels of parasites did not differ significantly between patients of groups A and B.

In group A, the parasitological evaluation demonstrated that cPCR and qPCR were positive in 40 (80 %) and 43 (86 %) of the cases, respectively, nevertheless XD was positive only in 10 cases (20 %). In group B, without cardiopathy, cPCR and qPCR were positive in 35 (70 %) and 35 (70 %) of the cases, respectively, nevertheless XD was positive only in 10 cases (20 %).

**Fig. 1:**
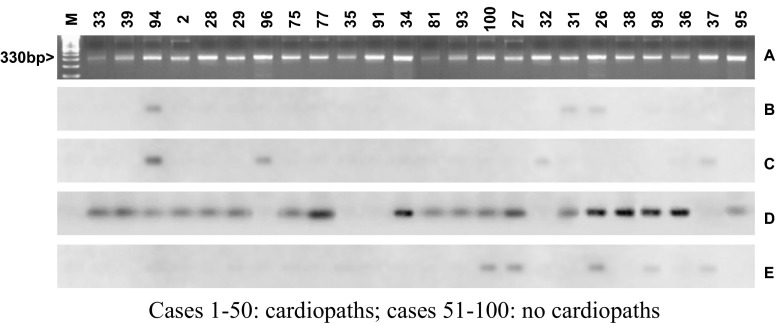
*Trypanosoma cruzi* cPCR amplicons stained with ethidium bromide (A). Hybridization profiles obtained with genotypes specific probes corresponding to TcI (B), TcII (C), TcV (D), and TcVI (E). The 330 base pair (bp) product represents a positive assay. Cases *1–50*: cardiopaths; cases *51–100:* no cardiopaths

The number of patients with positive qualitative and quantitative amplification of parasite DNA (cPCR and qPCR) was greater in group A compared to group B, 40 v/s 35 and 45 v/s 35 cases, respectively, but without statistical significance. Of the 100 individuals included in this study, 75 were cPCR positive. Of these, 45 were hybridized for genotyping. In the other 30 samples, *T. cruzi* DNA was insufficient for (21 samples) or were not recognized by the probes used (9 samples). According to representative results (Fig. 1), the most frequent *T. cruzi* DTU was TcV, but also TcI, TcII, TcVI, and mixtures up to three were circulating in peripheral blood (Table 3).

**Table 3:** Genotypes of *Trypanosoma cruzi* in 25 cardiopaths group A and 20 non-cardiopaths group B of Chile

		Group A	Group B
Infection	Single	18 (72 %)^a^	15 (75 %)^c^
	Mixed	7 (28 %)^b^	5 (15 %)^d^
Total		25	20

The most frequent DTU was the following: ^a^TcV (15 cases), ^b^TcV-TcVI-TcII (2 cases), ^c^TcV (10 cases), and ^d^TcV-TcVI (2 cases)

### Electrocardiographic and Echo-Doppler alterations of group A

The 50 cardiopaths of group A had 80 electrocardiographic alterations (Table 1). According to the NYHA classification, group A was divided into two sub-groups. In sub-group I, the 17 patients showed electrocardiographic alterations of grade I NYHA classification (1994; Andrade et al. 2011). There were 15 cases, 9 men and 6 women aged 38 to 81 years, who showed sinus bradycardia from 41 to 58 pm in the ECG tracing. In two others, one man of 46 and a woman of age 56 years right bundle branch block was incompletely blocked. In sub-group II, 14 women and 6 men had more than one electrocardiographic alterations. In eight cases, six women and two men aged 48 to 79 years had sinus bradycardia ranged. Three of these women had first-degree A-V block and prolonged QTc interval associated with bradycardia, in two cases and left ventricle hypertrophy in the others. One women had left anterior hemiblock, another had bradycardia in association with prolonged QTc interval, and the last woman showed ischemia in association with bradycardia. One man had left ventricle hypertrophy associated with bradycardia and the others prolonged QTc interval. Ten cases, 7 women and 3 men aged 49 to 77 years showed left anterior hemiblock, alone in 3 cases, one in association with left ventricle hypertrophy and in another two with prolonged QTc time, ventricular extrasystole and incomplete right bundle branch block, in association with prolonged QTc time in one case, in one with myocardial ischemia and auricular extrasystole, one with complete right bundle branch block and prolonged QTc interval and the last with sinus bradycardia (already mentioned). Prolonged QTc time appeared in 17 cases (12 women aged 31 to 79 years and 4 men aged 36 to 49 years). This was an isolated finding in three cases; in the other 14, it was associated. First A-V block was present in four patients, isolated in one, and the other three were associated (Table 1). Most of the patients of group A were in grade I or II of the NYHA classification. The Echo-Doppler was normal in 49 cases. One patient (number 21) with sinus bradycardia had a low ejection fraction (56 %) (Table 1). Gender and cardiopathy were not associated.

### Comorbility

In relation to comorbidity, 34 (68 %) patients of group A and 25 (50 %) of group B did have an associated pathology. This difference is not statistically significant (z = 1.43, p = 0.0764). The most frequent concomitant pathology in groups A and B was hypertension (58.8 and 48 %, respectively), followed by hypercholesterolemia in group A (29.4 %) and cholecystectomy in group B (36 %) (Tables 1 and 2).

## Discussion

The majority of the patients of group A (70 %) and group B (66.0 %) live in rural zones, where they have more contact with infected vectors and whose houses are appropriate for the development of the vectors (Briceño-León 1990; Schofield 1994; Gomes et al. 2013). This area is currently free of *T. infestans*, the most important domiciliary vector of Chagas disease (OPS/OMS 2000). Comparing the age groups (Tables 1 and 2) shows that there are more older people in group A (above 70 years) and more younger people in group B (under 30 years), and that the average age of group A, 57.8 years, is significantly greater than that of group B, 49.6 years. These results are consistent, since Chagas cardiopathy requires a prolonged period to develop (Elizari 1999; Rassi et al. 2009; Morillo 2013). In the present study, the cardiopathy increased with age. Females predominated in both groups (60 and 80 % group A and B, respectively) due to the greater attendance of women in clinical controls for Chagas disease, while their husbands were working and the greater willingness of women from endemic zones to transfer to the Metropolitan Region to perform cardiac Echo-Doppler. No association between sex and cardiopathy was observed; similar results were obtained by other investigators (Pereira et al. 1990; Silva et al. 2007), but are discordant with Basquiera et al. (2003), who reported more males with Chagas cardiomyopathy.

The results of the parasitological study of group A showed that the percentages of detection of *T. cruzi* by XD, cPCR, and qPCR were 20, 80, and 86 %, respectively. In the group B, the percentages were 20, 70, and 70 %, respectively. XD was positive in 20 % of groups A and B, concordant with literature where the sensitivity in chronic Chagas patients fluctuated between 5.3 and 50 % (Basso and Moretti 1984; Pereira et al. 1992; Siqueira-Batista et al. 1994). Three cases were XD (+) and PCR (−) (2 in group A and 1 in group B). Except for these, there was no discordance between XD and qPCR.

The results of sensitivity obtained by cPCR are also concordant with other studies on this period of the disease, with percentages between 43 and 75.2 % (Hidron et al. 2010; Gilber et al. 2013). In this study, the concordance between cPCR and qPCR in groups A and B was 78 and 84 %, respectively. The seven cases of group A and 4 of group B, with positive qPCR and negative cPCR, presented loads very close to the lower limit of the dynamic range established (between 0.009–0.04 parasites/ml). The quantification limit of qPCR for *T. cruzi* in our laboratory is 0.01 parasites/ml. Four cases of group A and four cases of group B had positive cPCR and negative qPCR. The sensitivity of kDNA cPCR in our laboratory with 5 ml of peripheral blood, if there is at least one intact parasite, the detection limit is 0.2 genomic equivalents (Schijman et al. 2003). This discordance was probably due to the use of different parasite targets, kDNA for cPCR and nuclear satellite DNA for qPCR (Moreira et al. 2013). No significant difference was found between average parasite burden in patients of group A and B, nevertheless in group B, there was a higher proportion of cases without parasites detectable by qPCR (15 against 7), and in group A, two cases were found with more than two parasites/ml of blood. This result differs from (Mosca et al. 1985) who demonstrated higher parasitemia by XD in cardiopaths than chronic chagasic patients without cardiopathy. It is also important to consider that parasitemia does not always represent the parasite burden; tissue parasite burden represents more exactly *T. cruzi* burden of chagasic patients (Vago et al. 2000; Valadares et al. 2008). Another study concluded that parasitemia does not correlate with the number of alterations detected in the electrocardiographic tracing (Tarleton and Curran 2012).

*T. cruzi* DTUs circulating in cardiopaths and non-cardiopaths appear to be the same. TcV was the most abundant and TcI the least frequent DTU detected in these patients, even though other not identified DTUs are also circulating in some patients. The presence of mixed DTUs (11/21) and undetermined DTUs (9/21) were also reported in a previous survey (Muñoz et al. 2013)

Our results agree with an experimental survey in mice inoculated with three main *T. cruzi* genotypes, in which all resulted with cardiomyopathy (De Diego et al. 1998) and TcV, the most frequent genotype in Argentina (Burgos et al. 2007; Cura et al. 2012). They differ from the genotypes more frequently observed in patients with cardiomyopathy, TcI in Colombia and Venezuela,, TcII in Brazil (Ramírez et al. 2010; Segovia et al. 2013) Therefore, ECG alterations are not related to the number or genotype of parasites. In addition, mild cardiopathies of Colombia and Argentina had 20 times higher parasite loads than patients from Brazil with severe cardiopathy (Moreira et al. 2013).

The diagnosis of Chagas cardiomyopathy was made based on clinical examination, results of the ECG and cardiac Echo-Doppler, which allowed ruling out the most important cardiomyopathies of other etiologies. In relation to comorbidity, hypertension was the most frequent pathology observed in both groups (Tables 1 and 2). In patients of group A who had hypertension and/or hypercholesterolemia, the cardiopathy was due to ChD and not to hypertension, as the ECG showed no left ventricular hypertrophy and the cardiac Echo-Doppler was normal. Group A had a greater co-morbidity (68 %) than group B (54 %); however, the difference is not statistically significant. Atherosclerosis was discounted as a confounding variable of the clinical data of heart pathology in the cohort, and no patients had a history of angina. The ECG analysis of the cohort showed no signs of ischemia and the cardiac Echo-Doppler was normal. In groups A and B, 6 and 9 women, respectively, had been cholecystectomized; this figure is not exceptional, because in Chile, 55 % of women over age 50 years have a gallbladder pathology (Braghetto et al. 2011). Two men of group A were also cholecystectomized, confirming that this pathology is also common in men. Ten patients had gastrointestinal involvement, three had esophagus achalasia, 1 of group A and 2 of group B, one patient of group B had megacolon, another a colon cancer, one had chronic gastritis, and the last a gastric ulcer. Three patients of group A had chronic gastritis. The electrocardiographic tracing was the basic clue for diagnosis of Chagas cardiomyopathy; 80 abnormalities were observed in the 50 cardiopaths. Sinus bradycardia was the most frequent alteration (23 cases, 28.5 %); in 15 patients, it was the only alteration of the serial ECG performed (at least three); none of these cases corresponded to athletes or people who do heavy work or received bradycardic vagotonic drugs that could cause this pathology. The eight associated cases correspond to the following: prolonged QTc interval (2), first-degree A-V block and prolonged QTc interval (2), one with LAHB, another with A-V first-degree block plus prolonged QTc interval and left ventricular hypertrophy; one case presented sinus bradycardia anterior ischemia and the last ventricular hypertrophy. The prolonged QTc interval is one of the first elements altered in the ECG of chagasic cardiopaths according our experience; this was present in 17 cases (21.25 %); in three of these it was the only alteration present. In the remaining 14, it was associated with sinus bradycardia (2), ventricular extrasystoles (2), sinus bradycardia plus first-degree A-V block (2), left anterior hemiblock (2), and complete right bundle branch block (2); the remaining four corresponded to left anterior hemiblock plus complete right bundle branch block, two to repolarization abnormalities and the last to systolic bradycardia plus left ventricle hypertrophy and first-degree A-V block. According to the electrocardiographic alterations, most of the 50 chagasic cardiopaths correspond to grade I, 17 cases (34 %) and grade II, 33 cases (66 %) of the NYHA classification; similar percentages were observed in Brazil (Rassi et al. 2010]. The most frequent electrocardiographic alterations of the group of cardiopaths were sinus bradycardia and prolonged QTc interval. The majority correspond to grades I and II of the NYHA classification. The parasite burden and genotypes of the group with Chagas heart disease and patients with indeterminate Chagas disease were similar.
